# Distant Phe345 mutation compromises the stability and activity of *Mycobacterium tuberculosis* isocitrate lyase by modulating its structural flexibility

**DOI:** 10.1038/s41598-017-01235-z

**Published:** 2017-04-21

**Authors:** Harish Shukla, Rohit Shukla, Amit Sonkar, Tripti Pandey, Timir Tripathi

**Affiliations:** Molecular and Structural Biophysics Laboratory, Department of Biochemistry, North-Eastern Hill University, Shillong, 793022 India

## Abstract

Isocitrate lyase (ICL), a potential anti-tubercular drug target, catalyzes the first step of the glyoxylate shunt. In the present investigation, we studied the conformational flexibility of MtbICL to better understand its stability and catalytic activity. Our biochemical results showed that a point mutation at Phe345, which is topologically distant (>10 Å) to the active site signature sequence (^189^KKCGH^193^), completely abolishes the activity of the enzyme. In depth computational analyses were carried out for understanding the structural alterations using molecular dynamics, time-dependent secondary structure and principal component analysis. The results showed that the mutated residue increased the structural flexibility and induced conformational changes near the active site (residues 170–210) and in the C-terminal lid region (residues 411–428). Both these regions are involved in the catalytic activity of MtbICL. Upon mutation, the residual mobility of the enzyme increased, resulting in a decrease in the stability, which was confirmed by the lower free energy of stabilization in the mutant enzyme suggesting the destabilization in the structure. Our results have both biological importance and chemical novelty. It reveals internal dynamics of the enzyme structure and also suggests that regions other than the active site should be exploited for targeting MtbICL inhibition and development of novel anti-tuberculosis compounds.

## Introduction

Proteins are intrinsically dynamic systems whose motions cover large ranges in both magnitude and timescale^1^. Thus, the structural dynamics and flexibility play an important role in the function of proteins. To obtain a detailed description and understanding of the function of a protein, the 3D structure and an accurate description of its dynamics are therefore required. Proteins interchange between structural states covering a magnitude from 10^−11^ to 10^−6^ m as well as spanning timescales from 10^−12^ s to 10^5^ s^2–4^. Structural biology nicely complements the experimental techniques to study such fast changes. In particular, molecular dynamics (MD) simulations have provided valuable understanding into protein dynamics at an atomic level in detail. From the ensemble of conformations, derived from MD simulations, or alternatively a large set of experimental structures, principal component analysis (PCA) can be performed^3, 5^. The resulting principal components (PCs) are sorted according to their contribution to the total fluctuation along the ensemble of conformations. This data can be used to study global, correlated motions in atomic simulations of proteins.

**Figure 1:**
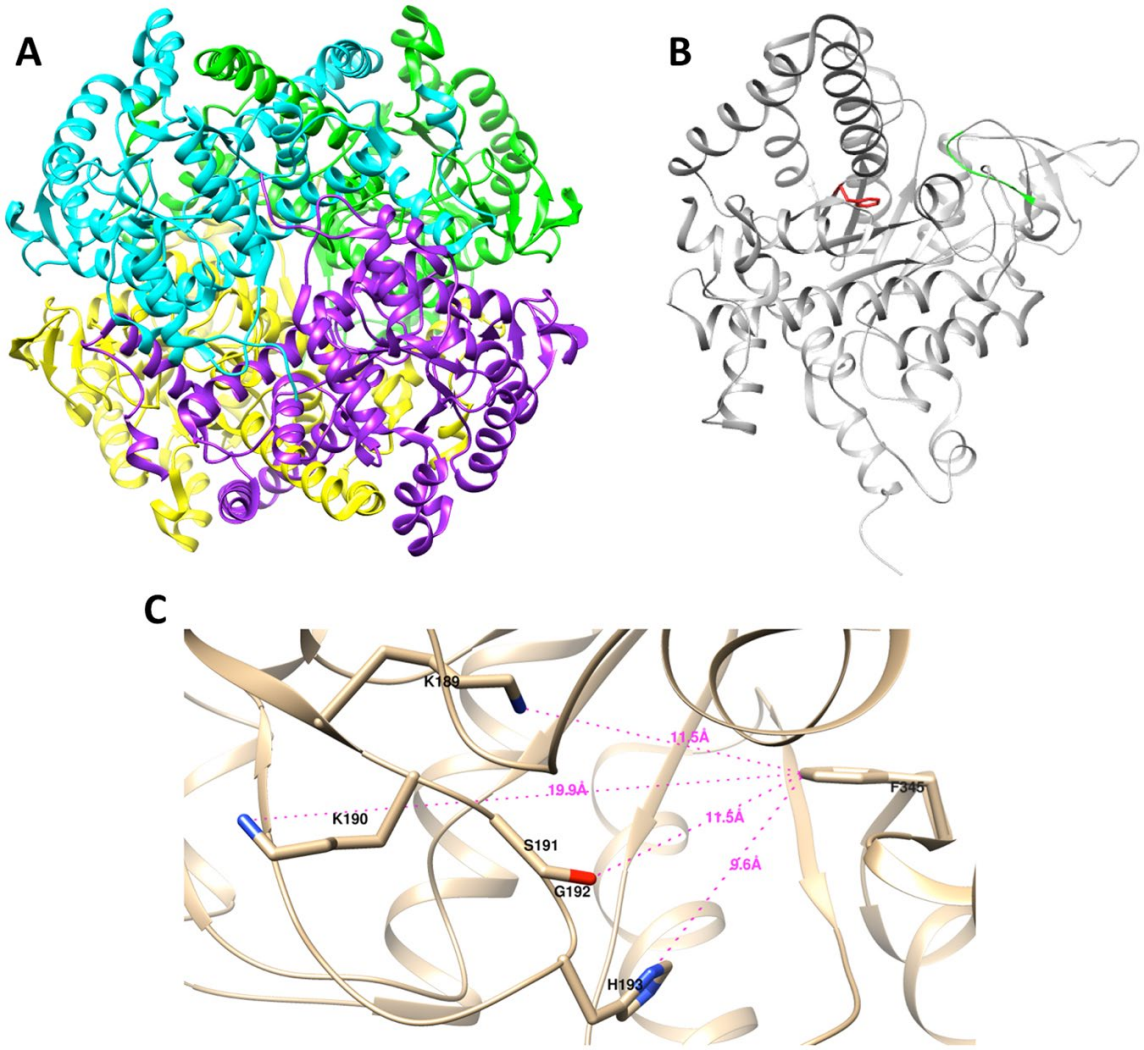
Structure of MtbICL. (**A**) Ribbon structure of the homotetrameric MtbICL, with each subunit shown in different color (PDB ID: 1F8I). (**B**) Chain A of MtbICL tetramer showing the position of Phe345 in red and active site signature sequence (^189^KKCGH^193^) in green. (**C**) Active site cavity of the chain A of MtbICL. The active site residues and Phe345 are marked and labeled. The distances are shown with dotted lines. The structures were visualized with UCSF Chimera.

Isocitrate lyase (ICL), one of the key enzymes of glyoxylate shunt, catalyzes the transformation of isocitrate to succinate and glyoxylate. It is important for carbon anaplerosis in the TCA cycle amid growth on C2 substrates such as fatty acids^6, 7^. The glyoxylate shunt is widespread among prokaryotes, lower eukaryotes and plants, but it is absent in vertebrates^8^. *Mycobacterium tuberculosis* requires beta-oxidation, gluconeogenesis and glyoxylate shunt to survive inside the phagosomes of macrophages, which are glucose deficient but fatty acid replete^9^. The ICL expression is upregulated in *Mycobacterium* during the infection of macrophages and the disruption of MtbICL inhibits the persistence of *M*. *tuberculosis* in the macrophage in mice^10–12^. Also, ICL has been reported to mediate broad antibiotic tolerance in *M*. *tuberculosis* ^13^. Hence, MtbICL is considered as one of the potential drug targets against persistent TB infection. The crystal structures of MtbICL are available in both apo-form and as complex with substrate analogues and inhibitors^10^. MtbICL is a tetrameric protein with each monomer containing 428 amino acids (Fig. 1A). Each subunit of MtbICL is composed of 14 α-helices and 14 β-strands. An unusual α/β barrel consisting of eight α-helices (α4–α11) and eight β-strands (β2-β5, β8, β12–β14) forms the larger domain of the enzyme. The helix α12 (residues 349–367) moves in a direction away from the barrel and then combines with two consequent helices, α13 (residues 370–384) and α14 (residues 399–409), to exclusively interact with the neighboring subunits. The small β-domain consisting of a short five-stranded β-sheet (β6, β7, β9–β11) and several of the active site residues lies on top of the α/β barrel. One remarkable characteristic of this structure is the inter-subunit helix swapping of α12 and α13 between two non-crystallographically related subunits that are responsible for the tetrameric structure. A similar feature has been reported in other proteins that help to form stable dimers^14^. Number of studies has been carried out to develop inhibitor of MtbICL, but no successful inhibitor has been found.

Alterations in amino acid sequence could affect the native 3D conformation of the protein structure and to estimate the conformational modifications, MD simulations can be used for an in-depth analysis. There are few reports that show the activity of an enzyme can be lost by a mutation topologically distant from active site. This is usually due to loss of structure and/or stability of the enzyme^15–17^. In the present study, we investigated the role of a topologically distant Phe345 in the structure-activity regulation of MtbICL. To achieve this goal, biochemical experiments and multi-dimensional computational studies were performed on MtbICL and MtbICL_F345A_ (hereafter referred as native and mutant protein respectively). Our results provide insights into how the F345A mutant altered its 3D structure leading to a complete loss of catalytic activity and correlate the structural changes with its activity owing to mutation. We also suggest that targeting regions other than the active site shall also be considered for the strategy to develop structure based inhibitor against MtbICL.

## Results and Discussion

### Preparation of recombinant native and mutant MtbICL

**Figure 2:**
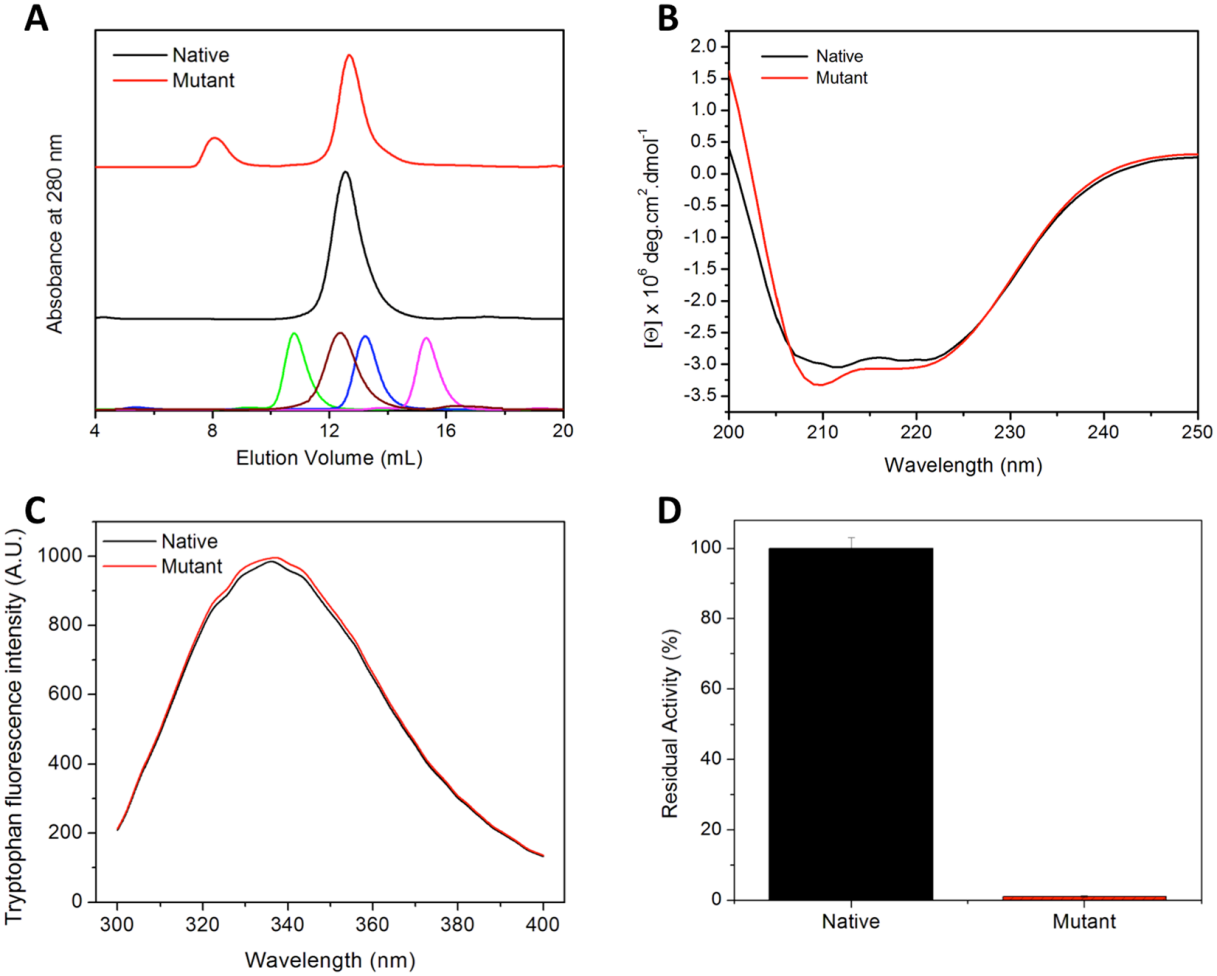
Comparative biochemical profiles of native and mutant MtbICL. (**A**) Size exclusion chromatography profiles. Lowest panel shows chromatogram of known standard molecular weight markers- ferritin (440 kDa at 10.8 mL, in green), catalase (232 kDa at 12.4 mL, in brown), aldolase (158 kDa at 13.2 mL, in blue) and ovalbumin (43 kDa at 15.3 mL, in pink). (**B**) Far-UV CD spectra, (**C**) Intrinsic tryptophan fluorescence emission curves and (**D**) Percent residual enzymatic activity.

The cloned and over-expressed native and mutant proteins were purified as described in the experimental section. The molecular weights of the purified recombinant proteins were determined by SDS-PAGE (Supplementary Fig. S1). On the Superdex^TM^ 200 column both the proteins showed peak with a retention volume of around 12.6 mL that corresponds to a molecular mass of ~200 kDa (Fig. 2A), as calculated from the values obtained for the protein standards. This suggests that both proteins existed as tetramer under normal conditions, however a small fraction of the mutated protein was found to form aggregate.

### Characterization of the secondary and tertiary structure of the native and mutant MtbICL

The secondary and tertiary structures of the proteins were probed using far-UV CD and intrinsic tryptophan fluorescence, respectively. The far-UV CD spectra of native and mutant proteins showed the presence of a typical α/β type secondary structure, with minor alterations (Fig. 2B). The tryptophan emission wavelength maximum for both native and mutant protein was observed around 336 nm (Fig. 2C) suggesting insignificant changes in tertiary structure between them. Both these results suggest that both the proteins are folded in proper conformation with minor alterations in their secondary structure.

### F345A mutation leads to complete loss of the enzyme activity

There are four highly conserved regions in the structure of ICL in all organisms, but only two of these regions are involved in the catalytic activity of the protein. The first conserved region involved in the activity contains an ICL signature sequence- ^189^KKCGH^193^, while the other is ^411^PNSSTTALTGSTEEGQFH^428^ ^10, 18, 19^. Both regions are thought to be important for substrate binding/catalysis as the non-homologous mutations in any of these regions in *E*. *coli* affect the activity of the protein^20–22^. We observed that a single amino acid mutation at position 345 (F345A), which is structurally distant from the active site signature sequence (^189^KKCGH^193^), leads to the complete loss of enzymatic activity in the mutant protein (Fig. 2D). This Phe345 is located at a distance of 10–12 Å from the active site signature sequence (^189^KKCGH^193^) of MtbICL (Fig. 1B and C).

### F345A mutation compromises the stability of the protein

**Figure 3:**
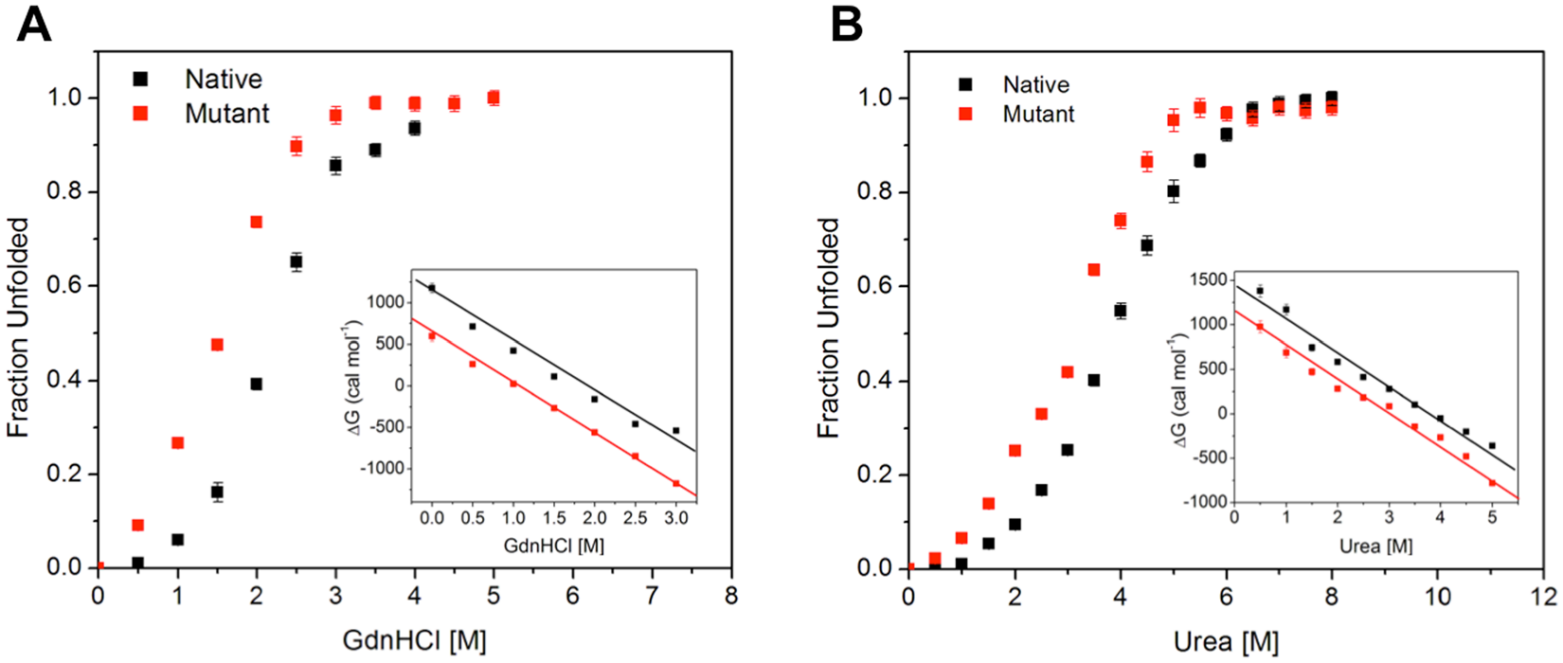
Determination of free energy of stabilization. Plot of fractional change in the wavelength maxima of the intrinsic tryptophan fluorescence of the native (black square) and mutant (red square) MtbICL with increasing concentration of (**A**) Urea and (**B**) GdnHCl. In both the figures, inset shows the linear free energy extrapolation curve of native (black square) and mutant (red square) MtbICL with respect to [Urea] and [GdnHCl] respectively. The ∆G^H20^ was the intercept on the Y-axis, obtained using the linear extrapolation method.

Equilibrium unfolding studies of a protein using chaotropic agents can provide the measure of its conformational stability^23^. Thus, urea and guanidine hydrochloride (GdnHCl)-induced denaturation studies were performed to determine the conformational stability of the native and mutant proteins using intrinsic tryptophan fluorescence (Fig. 3A and B). The denaturation curves showed a sigmoidal dependence, suggesting that both urea and GdnHCl-induced unfolding of native and mutant MtbICL was a two-state process. For proteins with a two-state transition, the *C*_m_ value (midpoint of the unfolding transition) serves as a probe for its stability against the denaturant^24, 25^. Interestingly, the *C*_m_ for mutant protein was considerably lower than that for the native protein, suggesting that the F345A mutation led to the destabilization of the protein structure (Table 1). The urea and GdnHCl-induced unfolding curves of native and mutant MtbICL were used to determine the free energy of stabilization in the absence of denaturants (ΔG^H20^) by linear extrapolation of the ΔG_D_ values to zero denaturant concentration (Fig. 3A and B inset)^24, 26^. The ΔG^H20^ value for native and mutant MtbICL were found to be approximately 1335 and 879 cal.mol^−1^ respectively for GdnHCl and 1432 and 1070 cal.mol^−1^ respectively for urea-induced denaturation, indicating that the native protein structure was comparatively more stable than the mutant protein (Table 1). These results suggest that Phe345 is involved in maintaining the structural stability of the protein.

**Table 1:** Conformational stability parameters of the native and mutant MtbICL.

Protein	Urea		GdnHCl	
	*C*_m_ [M]	ΔG^H20^ (cal.mol^−1^)	*C*_m_ [M]	ΔG^H20^ (cal.mol^−1^)
MtbICL	3.9	1432.9 ± 76.7	2.1	1335.3 ± 79.5
MtbICL_F345A_	2.9	1070.4 ± 64.2	1.5	879.9 ± 38.4

Unfolding studies were done at various concentrations of urea and GdnHCl as described in the Experimental section. The *C*_m_ and ΔG^H20^ values are based on three independent experiments for each measurement and mean ± SD was taken.

### F345A mutation modulates the structural dynamics and flexibility of MtbICL

To further understand the structural integrity of the proteins; we performed molecular dynamic simulation (MDS) studies. MDS is a method to find out the movement of atoms and molecules over a given period of time. Further, time-dependent secondary structure and principal component analysis (PCA) were carried out in detail to confirm the results.

#### F345A mutation induces the flexibility in the protein

**Figure 4:**
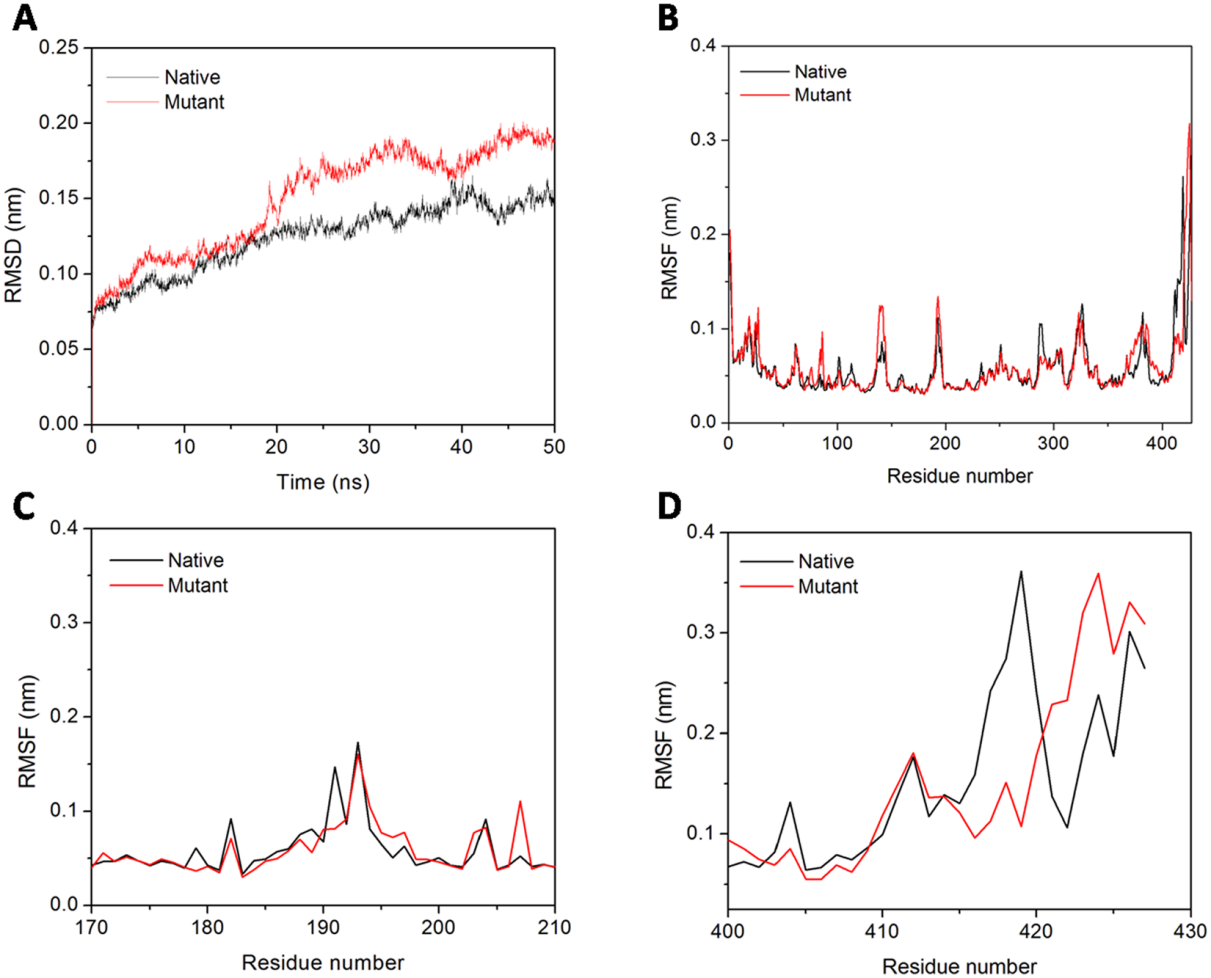
Molecular dynamic simulations. (**A**) RMSD of native and mutant MtbICL. (**B**), (**C**) and (**D**) represents the RMSF of the Cα atoms for chain A, active site region (residues 170–210) and C-terminal region (residues 400–427) of the native with mutant MtbICL respectively.

We then examined the effect of F345A mutation in the structural flexibility of the proteins *in silico* using MDS. The root mean square deviation (RMSD) and root mean square fluctuation (RMSF) were determined for native and mutant MtbICL. RMSD is used for measuring the difference between the backbones of a protein from its initial structural conformation to its final position. The stability of the protein relative to its conformation can be determined by the deviations produced during the course of its simulation. Smaller deviations indicate more stable protein structure. RMSD value for the Cα backbone was calculated for 50 ns simulation in order to evaluate the stability of both the systems. The RMSD profile indicated that during the initial periods of simulations, the native structure deviated considerably from the X-ray structure. Both systems were well equilibrated after 20 ns and produced stable trajectory for further analysis. The RMSD of native and mutant MtbICL are shown in Fig. 4A. Both proteins showed an average RMSD of 0.12 and 0.14 nm, respectively until the end of the simulations. The RMSD value indicates that the native protein was comparatively more stable than the mutant protein and that the F345A mutation influences the backbone stability of the protein leading to the destabilization of the structure.

To analyze the effect of mutation on the residue-wise mobility of the two proteins, we performed RMSF analysis of last 30 ns of the trajectory of native and mutant MtbICL. The RMSF graph (Fig. 4B) indicated that the mutant protein structure showed significantly higher fluctuations than the native protein structure. Major changes were observed near the active site (residues 170–210) and in the C-terminal region (residues 400 to 427) of mutant protein (Fig. 4C and D), suggesting the increased flexibility caused by the F345A mutation. Thus, an increase in the mobility of these regions might be responsible for the depletion in the catalytic activity in the mutant protein. It may be underlined that a decrease in rigidity and increase in overall flexibility was observed upon mutation, indicating that mutation cause structural alterations in the protein.

**Figure 5:**
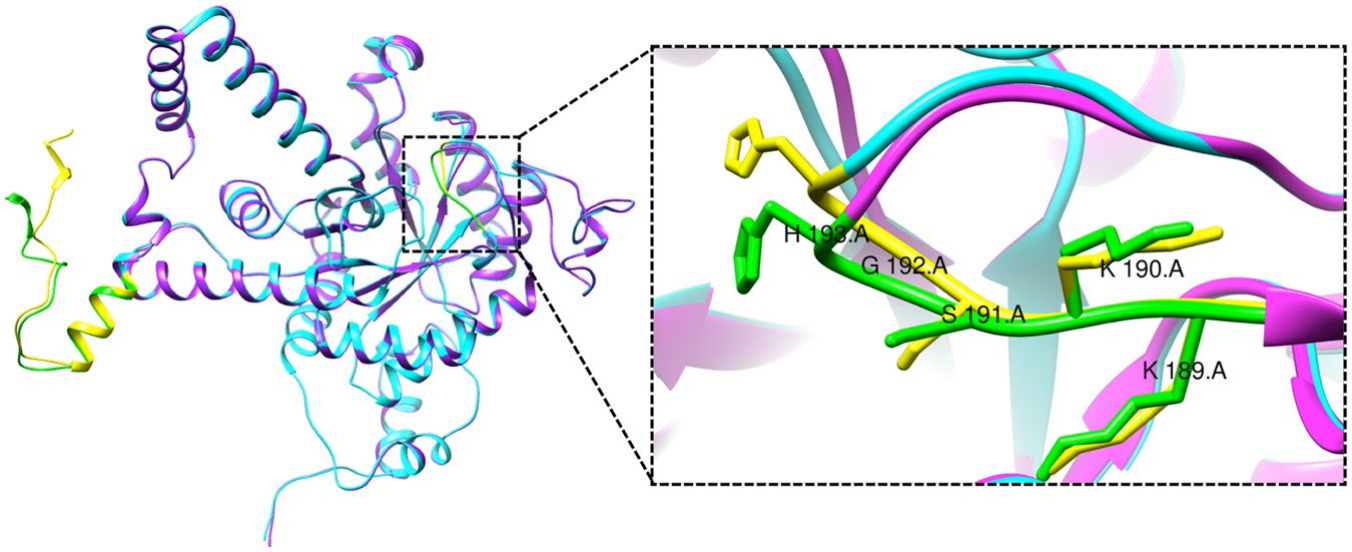
Average simulated structures. Superimposed average structure of the chain A of native (cyan) and mutant (pink) MtbICL. The active site signature sequence (^189^KKCGH^193^) and the C-terminal region (residues 400–427) are shown in yellow (native) and green (mutant). Inset shows the zoom view of the active site signature sequence (^189^KKCGH^193^) of native (yellow) and mutant (green) MtbICL.

All the frames were employed for generating the average structure using clustering approach for predicting the mobility of structural elements. Average structures for both native and mutant MtbICL were generated and used for structure alignment (Fig. 5). The RMSD value of this alignment was 0.535 Å.

#### Impact of F345A mutation on secondary structural elements and the active site pocket

The structural flexibility of a protein depends on the appearance of secondary structural elements during MD simulations. Secondary structure elements such as β-sheet and α-helix conformations are more rigid, while coils, bends, turns and 3-helix conformations are more flexible in nature. Time-dependent secondary structure fluctuations were calculated to predict additional information regarding the structural changes between native and mutant MtbICL of the last 30 ns of the trajectory. Changes in the secondary structure were observed for both proteins in comparison to each other. Secondary structure elements that were rigid in nature dominated in the native, while flexible secondary structure elements dominated in the mutant protein during simulations. The average structural changes were calculated for the whole trajectory and the results are shown in Supplementary Table S1. The results indicate that coil, β-bridge and α-helix conformation were decreased in mutant protein, whereas the β-sheets and bends were increased during simulation. To reduce the complex presentation of the four chains, the time-dependent secondary structure elements in the chain A are shown in Supplementary Figs S2 and S3 and the average changes are shown in Supplementary Table S2.

**Figure 6:**
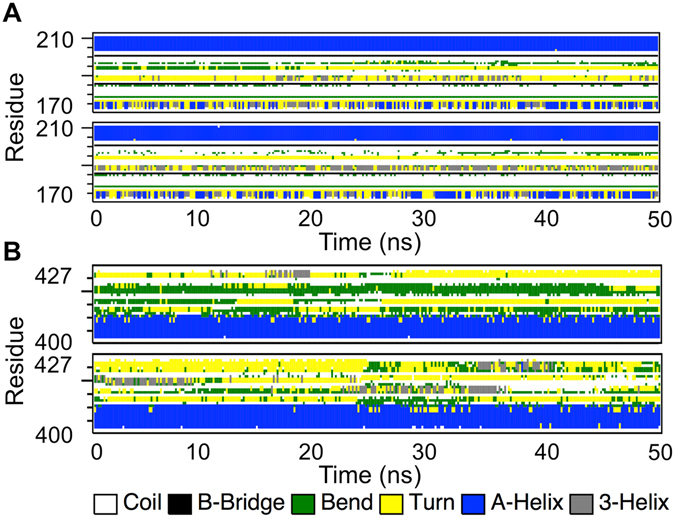
The time evolution of the secondary structural elements. The time evolution of the secondary structural elements of the native (upper panel) and mutant MtbICL (lower panel) of the (**A**) Active site region (residues 170–210) and (**B**) C-terminal region residues (residues 400–427).

We then checked the secondary structure changes near active site cleft (residues 170–210) as well as in the C-terminal region (residues 400–427) that showed significant changes (Fig. 6A and B). It was observed that in the catalytic cleft of native protein, the presence of bends, turns and few 3-helix was evident, but in the mutant protein the 3-helix formation was increased and the bend/turn formation was decreased that changed the conformation of catalytic cleft. Secondary structure changes between residues 400–427 showed that native protein structure has more number of coils while mutant protein has more number of turns, bends and α-helices. All these results suggest that the protein has lost its stability due to conformational changes after mutation. The secondary structural changes near the catalytic cleft (residues 170–210) and C-terminal region (residues 400–427) are shown in Supplementary Tables S3 and S4 respectively. The loss of rigid structural elements in the mutant and the presence of these elements in the native protein during simulation indicate the development of structural flexibility in the mutant protein. This structural behavior was further corroborated with PC analysis.

#### Principal component analysis confirms increased collective protein dynamics upon mutation

**Figure 7:**
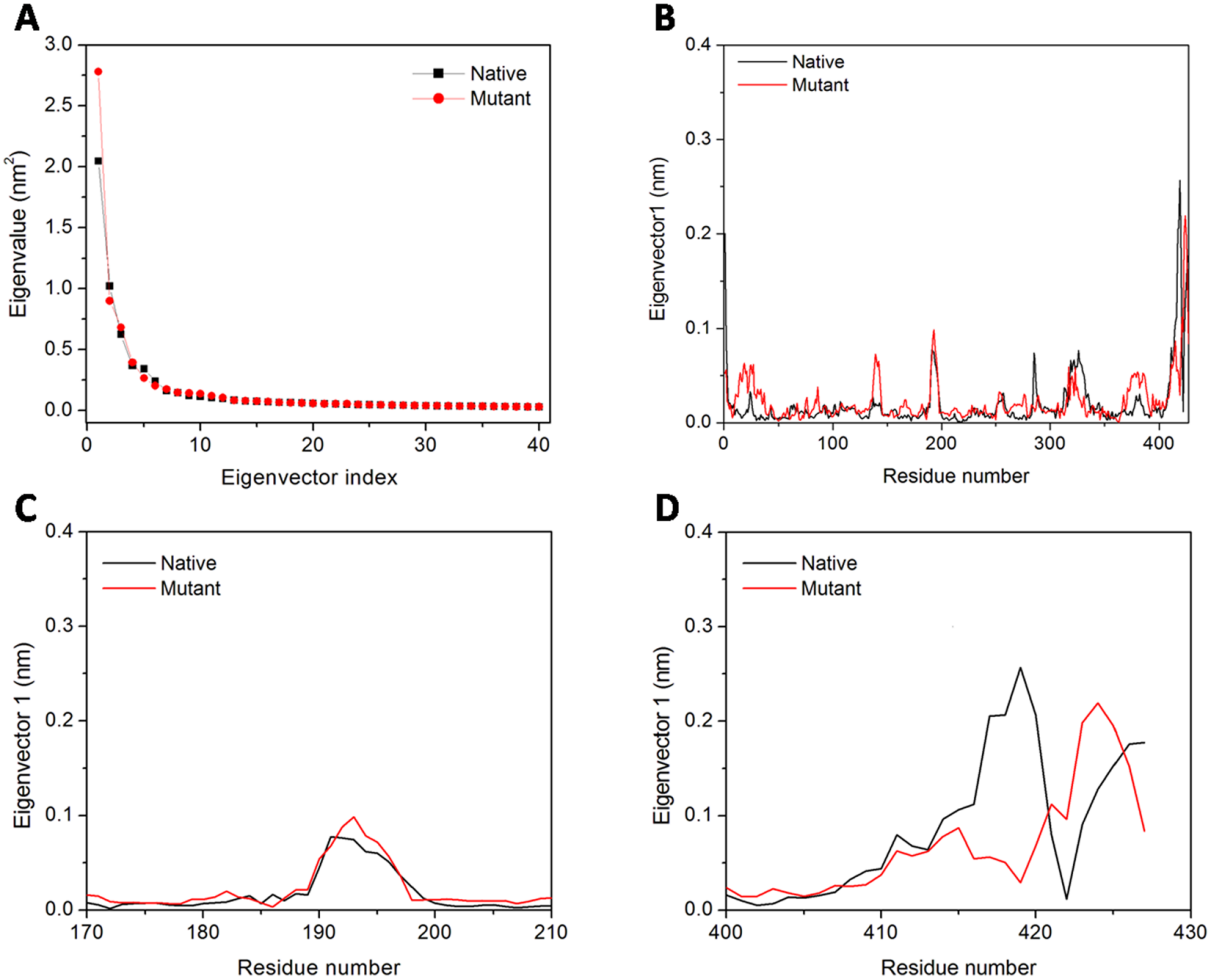
Principal component analysis. (**A**) The eigenvalues plotted against the corresponding eigenvector indices obtained from the Cα covariance matrix constructed from the last 30 ns of the MD trajectory. First 40 eigenvectors were used for calculations. (**B**, **C** and **D**) represents the displacements of the components of the chain A, active site region (residues 170–210) and C-terminal region (residues 400–427) of the native with mutant MtbICL respectively for the PC1.

To further support our MD simulation results, PCA were performed to predict the large-scale collective motions in native and mutant MtbICL and the dynamic mechanical properties of the systems. The principal component (PC) of protein is defined by the covariance matrix of the eigenvector and change in the particular trajectory along with each eigenvector obtained by this projection. Figure 7A shows a plot of the eigenvalues obtained from the diagonalization of the covariance matrix of the atomic fluctuations. The first few eigenvalues are relative to concerted motions and quickly decreased in amplitude to reach a number of constrained and more localized fluctuations. From this result, it was observed that the first 10 principal components (PC) account for 77.08% and 78.54% of the motions observed in the last 30 ns of the trajectories for native and mutant MtbICL respectively. This analysis suggests that the properties of the motions described by the first few PCs are different for both the systems. The magnitude of PC1 was increased by the point mutation. These observations thus validated the result of higher flexibility of the mutant compared to the native protein.

The dynamic behavior and level of fluctuation of the proteins were defined by the spectrum of corresponding eigenvalues and was confined within the first two eigenvectors. The projection of trajectories obtained onto the first two principal components (PC1, PC2) shows the motion of the two proteins in phase space. On these projections, we saw clusters of stable states. Two apparent features were observed from these plots. Firstly, in native protein, the cluster was well defined as compared to the mutant protein. Secondly, the mutant covered a larger region of phase space along the PC1 as well as the PC2 plane than the native (Supplementary Fig. S4). The native protein showed the spectrum range of PC1 and PC2 planes to be 2.13 nm and −2.56 nm, respectively, in the phase space while the mutant protein showed the range of PC1 plane as 2.25 and −2.90 nm in the PC2 plane. This result also shows that F345A mutation affects the native protein structure as the native spectrum occupies comparatively less space in the phase space while F345A mutation induces flexibility in the structure, so the mutant occupies more space in the phase space.

Ultimately, in order to understand how F345A mutation affects the motions described by PC1, the displacements of PC1 for both structures were calculated (Fig. 7B). Figure 7C and D suggests that F345A mutation can influence the displacements of the components near the active site (residues 170–210) and in the C-terminal region (residues 400–427) of protein, which is consistent with the RMSF analysis (Fig. 4C and D).

### Molecular docking of nitropropionate with native and mutant simulated MtbICL structure

**Figure 8:**
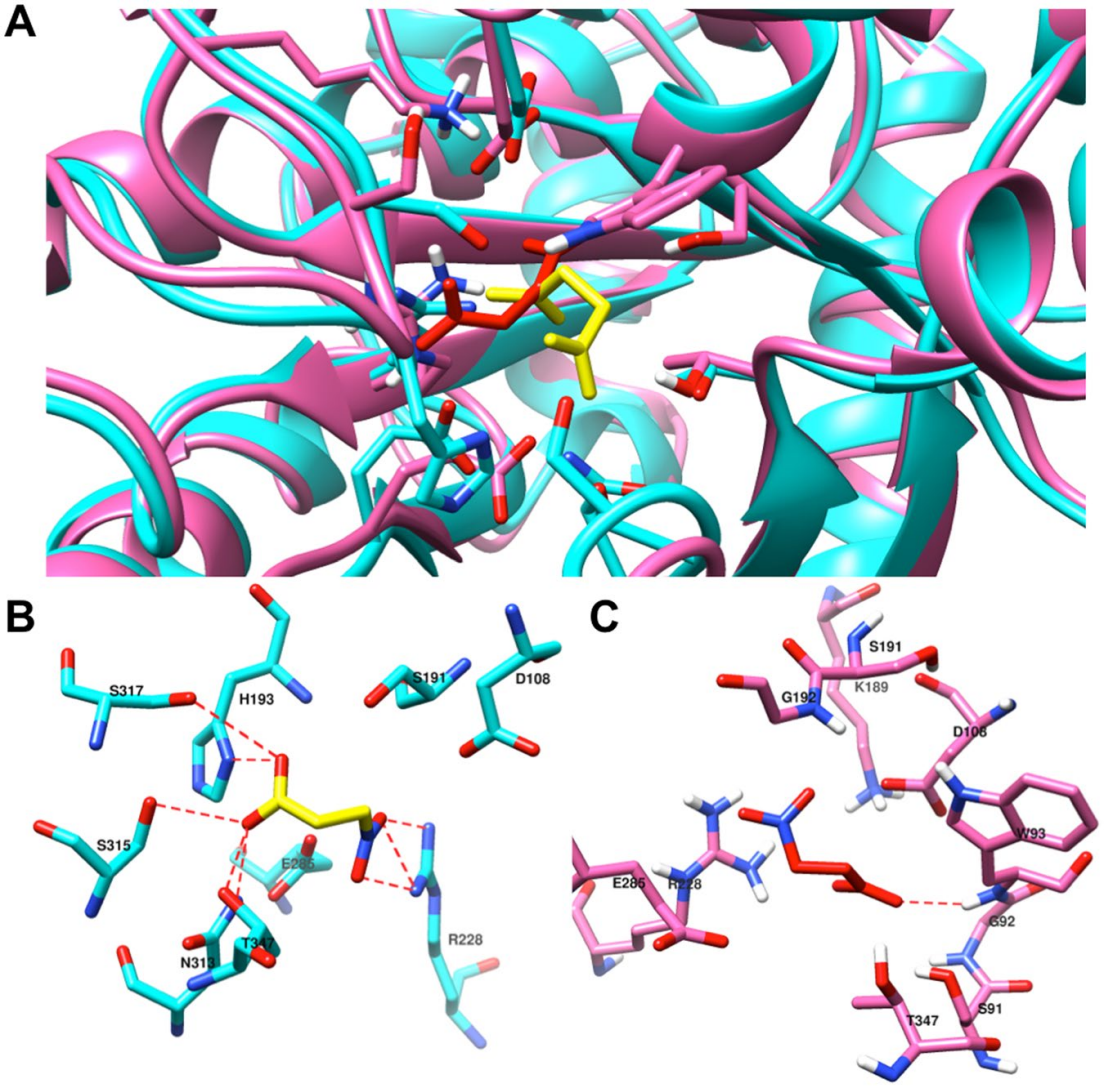
Molecular docking of 3-nitropropionate with native and mutant MtbICL. (**A**) Structure alignment of docked native and mutant (from last 50 ns simulation) MtbICL was carried out using Chimera 1.10.2. Native and mutant MtbICL are shown in cyan and pink colours respectively. 3-Nitropropionate for native and mutant MtbICL is shown in yellow and red colour respectively. (**B**) Position of 3-nitropropionate in the binding pocket of the native MtbICL. Structure of 3-nitropropionate is shown in yellow colour and stick form. The residues involved in bonding and non-bonding with 3-nitropropionate are shown in cyan colour. The dashed red lines represent the hydrogen bonding interaction between 3-nitropropionate and amino acid residues- His193 (3.15 Å), Arg228 (3.00 Å) and Asn313 (3.13 Å), Ser315 (3.00 Å) and Thr347 (3.09 Å) from A chain at the catalytic site of native MtbICL. (**C**) Position of 3-nitropropionate in the binding pocket of the mutant MtbICL. Structure of 3-nitropropionate is shown in red colour and stick form. The residues involved in bonding and non-bonding with 3-nitropropionate are shown in pink colour. The dashed red lines represent the hydrogen bonding interactions between 3-nitropropionate and amino acid residues- Trp93 (2.36 Å) from A chain at the catalytic site of mutant MtbICL.

The competitive inhibitor 3-nitropropionate was docked to both the native and mutant MtbICL structure. It was observed that the native MtbICL forms a total of eight hydrogen bonds with the inhibitor while the mutant forms only one hydrogen bond (Fig. 8). Also, the binding energy of 3-nitropropionate was more in case of native (−5.7 Kcal.mol^−1^) as compared to mutant (−4.2 Kcal.mol^−1^). These results further confirm that the catalytic activity of the enzyme was lost upon mutation due to distortion of the active site geometry.

## Conclusion

The structural dynamics and inhibition associated with MtbICL can form an important platform for rationalizing the structure-based drug discovery against *M*. *tuberculosis* as well as other microorganisms as the glyoxylate cycle is absent in humans whereas most of the bacteria and other parasites had this pathway which functions mainly in absence of TCA cycle. In addition to hundreds of scientists across world looking for inhibitor of MtbICL, Glaxo-SmithKline (in collaboration with Global TB Alliance) and Tuberculosis Antimicrobial Acquisition and Coordinating Facility of NIH^27–29^ have carried out extensive high throughput screening studies of a number of compounds but no successful inhibitor was found. Certain molecules including salicylanilide, benzanilide, 3-nitropropionamide, pthalazinyl derivatives, pyruvate-isoniazid analogs and its copper complexes are among the synthesized compounds showing a great potential to inhibit mycobacterial ICL and a significant anti-mycobacterial effect. Though these compounds need to be investigated further to understand their mechanism of inhibition of ICL activity before bringing them to clinical trials. Our results suggest that inhibitor screening should also be performed for regions other than the active site pocket of MtbICL, as disruption of other regions can also inhibit the activity of MtbICL.

## Methods

### Cloning and site-directed mutagenesis

The cloning of full-length MtbICL has been described previously and was a kind gift from Late Dr. Vinod Bhakuni, CDRI, Lucknow^30^. The mutant ICL, MtbICL_F345A_, was generated using the GeneTailor™ Site-Directed Mutagenesis System (Invitrogen) and mutagenic primer pairs 5′CCATGGGCTTCAAGTTTCAGGCAATCACGCTGGC3′ and 5′CTGAAACTTGAACCCCATGGCTGCCAGCTCC3′. The DNA sequence of the mutated gene was confirmed using sequencing.

### Preparation of recombinant proteins

MtbICL expression was carried out as described previously^30^. The expression of mutant MtbICL was performed similarly as that of native MtbICL. The purity of the recombinant proteins was checked by SDS-PAGE. Gel filtration experiments were carried out on a Superdex^TM^ 200 10/300 GL column (manufacturer’s exclusion limit 600 kDa for proteins) on an ÄKTA-FPLC (GE HealthCare Biosciences, USA) to determine the oligomeric status and molecular weight of the proteins. The column was pre-equilibrated with standard molecular weight markers and run with 50 mM phosphate buffer (pH 8.0), containing100 mM NaCl, at a flow rate of 0.5 mL/min at 25 °C.

### Activity assays

The activity of recombinant proteins was measured at 30 °C in 20 mM HEPES buffer, pH 7.5, using a modified continuous method as described previously^18, 31^. The reaction mixture also contained 5 mM MgCl_2_, 4 mM phenylhydrazine hydrochloride, 30 mM *threo*-dl-isocitrate and 1 mL enzyme. 25 nM enzyme was added to start the reaction and the cleavage of isocitrate was measured by the change in absorbance at 324 nm that resulted in glyoxylatephenylhydrazone (ε_324_ nm = 17000 M^−1^ cm^−1^).

### Fluorescence and circular dichroism (CD) measurements

Tryptophan fluorescence spectra were recorded with a Perkin Elmer Life Sciences LS 55spectrofluorimeter in a 5 mm path length quartz cell at 25 °C. An excitation wavelength of 280 nm was used and the spectra were recorded between 300 and 400 nm. 3 µM protein was used for the studies. CD measurements were made on JASCO J810 spectropolarimeter calibrated with ammonium (β)-10-camphorsulfonate with a 2 mm path length cell at 25 °C. The mean residual ellipticity [θ], was calculated as $[\theta ]=\frac{{\theta }_{obs}}{10\,\times \,lc}$, where *θ*_obs_ is the observed ellipticity in mdeg, c is the concentration in moles per liter, and l is the path length of the cuvette in cm. Five consecutive scans were accumulated and the average spectra stored. The values obtained were normalized by subtracting the baseline recorded for the buffer under similar conditions. All Data were fitted using Origin 7.0 Software.

### Calculation of free energy of stabilization

3 µM native or mutant proteins were dissolved in 50 mM phosphate buffer, pH 8.0 in the presence of increasing concentration of GdnHCl or urea and incubated overnight at 25 **°**C to achieve equilibrium before the tryptophan fluorescence measurements. By assuming a two-state model of protein unfolding, the denaturant-induced unfolding curves were used to determine the free energy of stabilization in the absence of denaturants (ΔG^H20^). For this, the ΔG_D_ values were linearly extrapolated to zero denaturant concentration as described previously^24, 26^. The experiments were repeated thrice and mean ± SD was taken.

### Molecular dynamics simulation

The structure of tetrameric MtbICL was obtained from the Protein Data Bank (PDB ID: 1F8I). MDS was performed using GROMACS 4.6.5^32, 33^ on an in-house supercomputer as earlier^34, 35^. Native and mutant ICL were solvated in a cubic box using TIP3P water model. The topology of the proteins was generated by using Amber ff99SB force field^36^. The systems showed −72 negative charges; thus, 72 Na^+^ ions were added for neutralizing the systems using the genion tool. All systems were subjected to steepest energy minimization to give the maximum force below 1000 kJ.mol^−1^ nm^−1^ to remove steric clashes of the systems. Long-range electrostatic forces were calculated by the Particle Mesh Ewald method^37^. A 1.0 nm radius cut-off was used for the computation of Lennard-Jones and Coulomb interactions. Bond lengths were constrained using the LINCS algorithm^38^. All bonds including H-bonds were fixed using the Shake algorithm. After energy minimization, the systems were equilibrated. Then the position restraint simulation of 1 ns was carried out under NVT (the constant Number of particles, Volume and Temperature) and NPT (the constant Number of particles, Pressure and Temperature) conditions. Finally, both the systems were submitted to 50 ns MDS. A 2 fs interval was given for saving the coordinates. The RMSD and RMSF were calculated using g_rms and g_rmsf tools. Time dependent secondary structures were calculated using do_dssp tool. Finally, the trajectory was analyzed by Visual Molecular Dynamics^39^ and Chimera^40^. GRACE and Origin softwares were used for generating and visualizing the plots (http://plasma-gate.weizmann.ac.il/Grace).

### Principal component analysis

PCA was used to calculate eigenvectors, eigenvalues and their projection along the first two principal components of both native and mutant MtbICL. The PCA method was embedded in the GROMACS software package. The concerted motions of macromolecules were extracted during simulation by PCA that are meaningful for biological function^41^. Rotational and translational movements were removed from the trajectory used in the construction of variance/co-variance matrix. The positional covariance matrix C of atomic coordinates and its eigenvectors were used. The elements of the positional covariance matrix C were calculated by the following equation:

1 $${C}_{i}=({q}_{i}-\langle {q}_{i}\rangle )({q}_{j}-\langle {q}_{j}\rangle )\,(i,j=1,2,\ldots ,3N)$$

where *q*_*i*_ is the Cartesian coordinate of the *i* ^th^ Cα atom and N is the number of Cα atom in native and mutant MtbICL. The average was calculated over the equilibrated trajectory after superimposition on a reference structure to remove overall translations and rotations by using a least-square fit procedure. The matrix was diagonalized by an orthogonal coordinate transformation matrix *Λ*, for predicting the set of eigenvectors and eigenvalues *λ*_*i*_:

2 $${\rm{\Lambda }}={T}^{T}{C}_{ij}T$$

where the columns are the eigenvectors corresponding to the direction of motion relative to <*q*_*i*_> and each eigenvector associated with an eigenvalue that represented the total mean-square fluctuation of the system along the corresponding eigenvector. The last 30 ns production runs were used to perform the analysis. The amplitude of eigenvector was represented by eigenvalues in the multidimensional space. The movement of atoms along each eigenvector indicated the protein’s concerted motions along each direction. The Cartesian trajectory coordinates were projected along the most important eigenvectors to identify the movements of structures in the essential subspace. Cα was selected for calculation. The g_anaeig and g_covar tools were used for PCA.

### Molecular docking

To predict the binding mode of 3-nitropropionate with native and mutant MtbICL, the simulated structures were docked against 3-nitropropionate using Autodock Vina^42^ in the identified binding cavity and the best pose was selected for visualization.

## Electronic supplementary material

Supplementary Information
