# Supplementary material for: Distant Phe345 mutation compromises the stability and activity of Mycobacterium tuberculosis isocitrate lyase by modulating its structural flexibility
Source: Sci Rep. 2017 Apr 21;7:1058. doi: 10.1038/s41598-017-01235-z (PMC5430663; doi:10.1038/s41598-017-01235-z)
Supplement: Supplementary file 1 — Supplementary Information [file 41598_2017_1235_MOESM1_ESM.pdf]

Supplementary Material

Distant Phe345 mutation compromises the stability and activity of *Mycobacterium tuberculosis* isocitrate lyase (MtbICL) by modulating its structural flexibility

Harish Shukla[#], Rohit Shukla[#], Amit Sonkar[#], Tripti Pandey, and Timir Tripathi

Molecular and Structural Biophysics Laboratory, Department of Biochemistry, North-Eastern Hill University, Shillong 793022, India

Running title: Conformational dynamics of inactive $MtbICL_{F345A}$ *mutant*

To whom correspondence should be addressed: Dr. TimirTripathi, Department of Biochemistry, North-Eastern Hill University, Shillong- 793022, India. Email: timir.tripathi@gmail.com, ttripathi@nehu.ac.in; Tel: +91-364-2722141; Fax: +91-364-2550108.

[#] These authors contributed equally to the work.

Supplementary Fig. S1. SDS-PAGE showing the purified native and mutant MtbICL. Lanes 1-3 represents molecular weight markers, native and mutant MtbICL respectively.

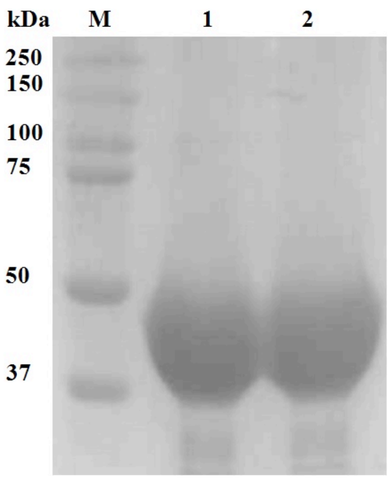

Supplementary Fig. S2. The time evolution of secondary structural elements changes in the chain A of native MtbICL.

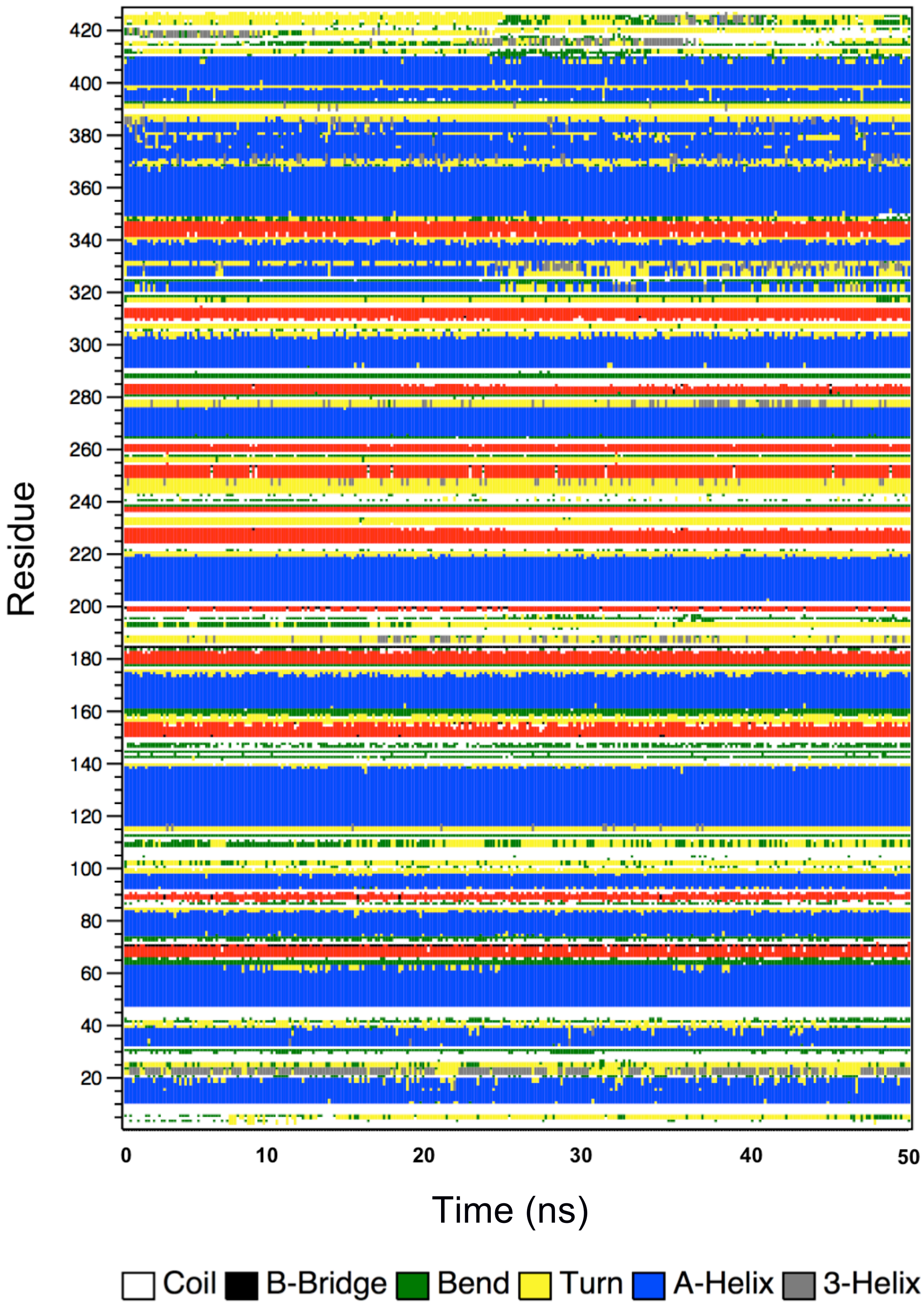

Supplementary Fig. S3. The time evolution of secondary structural elements changes in the chain A of mutant MtbICL.

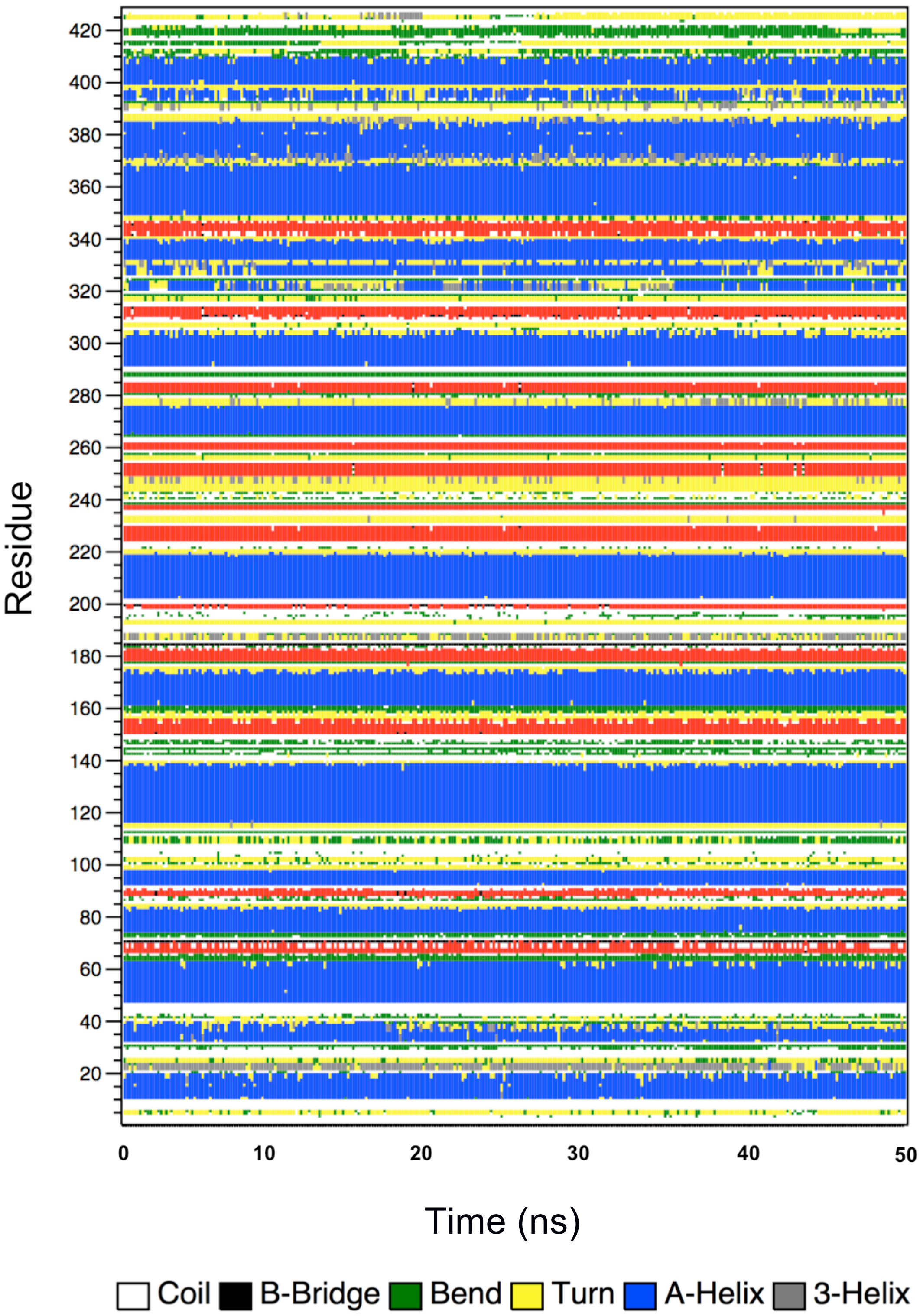

Supplementary Fig. S4. Projection of the motion of the protein in phase space along the first two principal eigenvectors for native and mutant MtbICL.

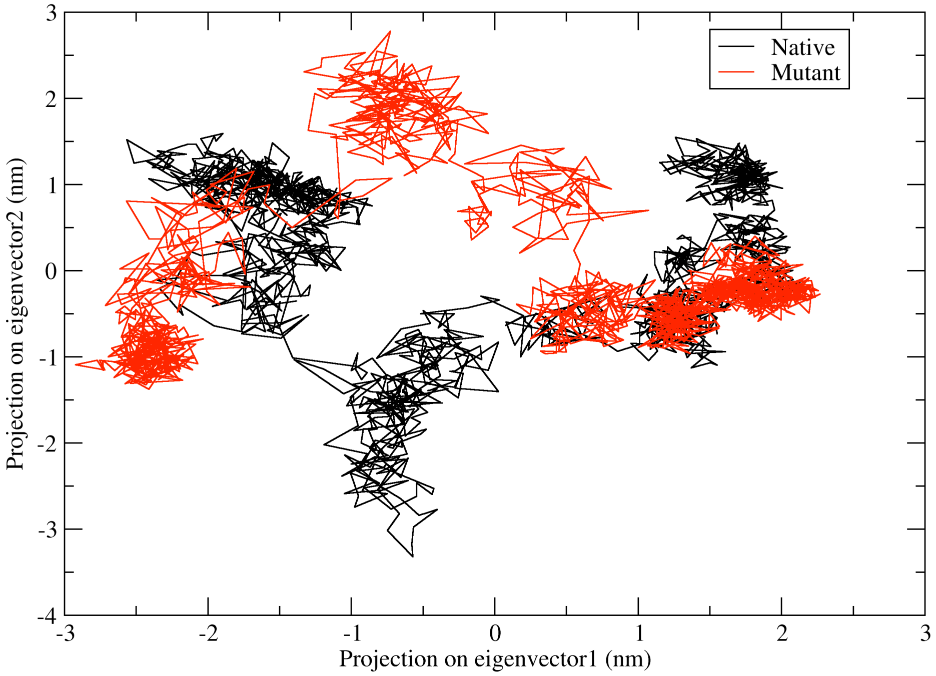

Supplementary Table S1. Time dependent average secondary structure element changes for tetrameric native and mutant MtbICL.

Name	Structure	Coil	β-Sheet	β-Bridge	Bend	Turn	α-Helix	5-Helix	3-Helix
**Native**	355481 (0.69%)	103522 (0.20%)	56821 (0.11%)	2688 (0.01%)	44343 (0.09%)	77105 (0.15%)	218867 (0.42%)	10762 (0.02%)	903 (0.00%)
**Mutant**	353129 (0.69%)	103015 (0.20%)	57737 (0.11%)	2466 (0.00%)	45796 (0.09%)	74933 (0.15%)	217993 (0.42%)	12168 (0.02%)	903 (0.00%)

Supplementary Table S2. Time dependent average secondary structure element changes for chain A of native and mutant MtbICL.

Name	Structure	Coil	β-Sheet	β-Bridge	Bend	Turn	α-Helix	5-Helix	3-Helix
**Native**	90163 (0.70%)	25876 (0.20%)	14324 (0.11%)	626 (0.00%)	10417 (0.08%)	20120 (0.16%)	55093 (0.43%)	-	2071 (0.02%)
**Mutant**	88308 (0.69%)	25727 (0.20%)	13946 (0.11%)	683 (0.00%)	11642 (0.09%)	18779 (0.15%)	54900 (0.42%)	-	2850 (0.02%)

Supplementary Table S3. Time dependent average secondary structure element changes in the active site region (170-210 amino residues) of native and mutant MtbICL.

Name	Structure	Coil	β-Sheet	β-Bridge	Bend	Turn	α-Helix	5-Helix	3-Helix
**Native**	5314 (0.43%)	5661 (0.46%)	na	601 (0.05%)	937 (0.08%)	1829 (0.15%)	2883 (0.23%)	na	429 (0.03%)
**Mutant**	5124 (0.42%)	5812 (0.47%)	na	602 (0.05%)	655 (0.05%)	1567 (0.13%)	2955 (0.24%)	na	750 (0.06%)

Supplementary Table S4. Time dependent average secondary structure element changes in C-terminal region (400-427 amino residues) of native and mutant MtbICL.

Name	Structure	Coil	β-Sheet	β-Bridge	Bend	Turn	α-Helix	5-Helix	3-Helix
**Native**	4539 (0.54%)	2579 (0.31%)	na	na	953 (0.11%)	2001 (0.24%)	2538 (0.30 %)	na	357 (0.04%)
**Mutant**	3887 (0.46%)	2577 (0.31%)	na	na	1904 (0.23%)	1413 (0.17%)	2474 (0.29%)	na	60 (0.01%)
